# Transcription factor VvibHLH93 negatively regulates proanthocyanidin biosynthesis in grapevine

**DOI:** 10.3389/fpls.2022.1007895

**Published:** 2022-08-24

**Authors:** Jing Cheng, Ying Shi, Jun Wang, Changqing Duan, Keji Yu

**Affiliations:** ^1^Center for Viticulture and Enology, College of Food Science and Nutritional Engineering, China Agricultural University, Beijing, China; ^2^Key Laboratory of Viticulture and Enology, Ministry of Agriculture and Rural Affairs, Beijing, China; ^3^Jiangsu Agri-Animal Husbandry Vocational College, Taizhou, Jiangsu, China

**Keywords:** grape, leucoanthocyanidin reductase, regulation, bHLH transcription factor, proanthocyanidin

## Abstract

Proanthocyanidins (PAs) derived from grape berries determine the astringency and bitterness of red wines. The two leucoanthocyanidin reductases (VviLAR1 and VviLAR2) are crucial for PA accumulation in grapevine. Our previous studies show that the promoter of *VviLAR1* contains multiple proposed bHLH transcription factor binding sites, but the corresponding bHLH family regulators remain unknown. Here we identified and functionally characterized VvibHLH93 as a new bHLH transcription factor in PA pathway. Yeast one-hybrid and electrophoretic mobility shift assays showed that VvibHLH93 bound the E/G-box in *VviLAR1* promoter. And *VvibHLH93* gene was mainly expressed in grape flowers, tendrils, stems and berries at PA active stages. Overexpression of *VvibHLH93* suppressed PA accumulation in grape callus, which was linked to the repression of the transcript levels of two *VviLAR*s. The gene expression analysis in transgenic grape callus and the dual-luciferase assay in tobacco leaves together revealed that VvibHLH93 targeted a broad set of structural genes and transcription factors in flavonoid pathway. This research enriches the regulatory mechanism of the two *VviLAR* genes, and provides new insights into regulating PA content in grape berries.

## Introduction

Grapevine (*Vitis vinifera* L.) is one of the most widely cultivated horticultural plants worldwide with important economic values, producing a significant level of proanthocyanidins (PAs, also named condensed tannins) in berries, leaves, and flowers (Wei et al., 2020). PAs maintain the yield and quality of grape berries by enhancing fungal resistance and repelling herbivores (Bogs et al., 2005; Dixon et al., 2005). Grape-derived PAs contribute to astringent and bitterness properties of red wines, and their co-pigmentation with anthocyanins can also improve the color stability during wine aging (Ma et al., 2014). In addition, PAs possess antioxidant capacity and radical scavenging function, which are beneficial to human health (Cos et al., 2004; Butelli et al., 2008). Therefore, understanding the biosynthesis and regulation of PAs in grapevine is important for further manipulating the polyphenol trait of grape berries.

**Figure 1:**
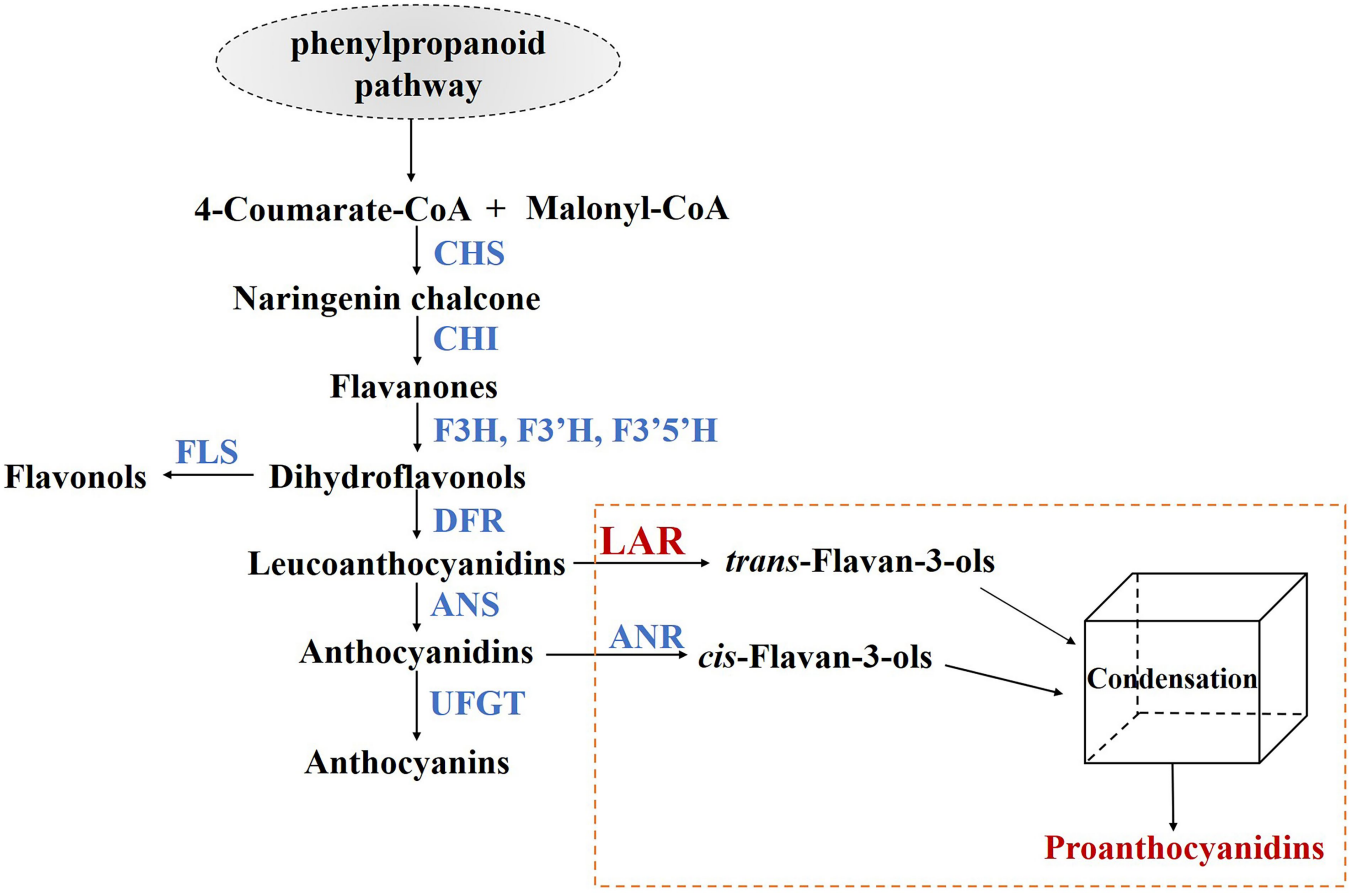
PA biosynthesis pathway in grapevine. CHS, chalcone synthase; CHI, chalcone isomerase; F3H/F3′H/F3′5′H, flavanone 3/3′/3′5′-hydroxylase; FLS, flavonol synthase; DFR, dihydroflavonol 4-reductase; ANS, anthocyanin synthase; LAR, leucoanthocyanidin reductase; ANR, anthocyanidin reductase; UFGT, UDP-glucose: flavonoid-3-*O*-glucosyltransferase.

PA biosynthesis branch belongs to flavonoid pathway, sharing several common upstream steps with anthocyanin and flavonol products (Figure 1; Dixon et al., 2005; Li et al., 2016a). At the entrance of PA and anthocyanin biosynthesis branches, dihydroflavonol 4-reductase (DFR) converts dihydroflavonols to the corresponding leucoanthocyanidins (Martens et al., 2002), which are substrates of anthocyanidin synthase (ANS) to produce anthocyanidins (Saito et al., 1999). In grapes, two leucoanthocyanidin reductases (VviLAR1 and VviLAR2) and anthocyanin reductase (VviANR) are key enzymes for synthesizing PA building blocks (Bogs et al., 2005). VviANR functions downstream of ANS and competes with UDP-glucose:flavonoid-3-*O*-glucosyltransferase (UFGT) to catalyze anthocyanidins to 2,3-*cis*-flavan-3-ols (commonly (−)-epicatechin). *VviANR* gene is expressed throughout flowering and berry development until veraison in grapevine (Bogs et al., 2005). VviLAR1 and VviLAR2 possess similar functions of converting leucoanthocyanidins into 2,3-*trans*-flavan-3-ols (commonly (+)-catechin) and regulating the degree of polymerization of PAs (Yu et al., 2019). Although (−)-epicatechin is the predominant PA subunits in grapevine, the knockout of a single *VviLAR1* gene is sufficient to reduce PA content in transgenic grape berries (Robinson et al., 2021). The expression of *VviLAR1* is restricted in flowers, young berries, and seeds, while *VviLAR2* transcript is mainly detected in berry skins before veraison, seeds during veraison, and expending leaves (Bogs et al., 2005). In addition, previous studies show that the expression of *VviLAR1* and *VviANR* is light-inducible, whereas the promoter activity of *VviLAR2* is not sensitive to the change of light intensity (Koyama et al., 2012; Sun et al., 2017, 2019; Cheng et al., 2020). The above findings imply that different regulation mechanisms are underlying the expression of the three PA-specific genes in grapevine.

Multiple transcription factors belonging to MYB, bHLH, and WD40 families have been characterized in grapevine, among which MYB regulators are intensively studied (Rousserie et al., 2019; Wei et al., 2020). Deluc et al. showed that VviMYB5a and VviMYB5b promote PA production by activating the upstream flavonoid pathway (Deluc et al., 2006, 2008). And the follow-up studies sequentially identified VviMYBPA1, VviMYBPA2 and VviMYBPAR are the three transcription activators mainly target to PA-specific genes *VviLAR1* and *VviANR* (Bogs et al., 2007; Terrier et al., 2009; Koyama et al., 2014). Later, Huang et al. found VviMYBC2-L1 suppresses PA biosynthesis by downregulating *VviMYBPA1*, *VviMYBPA2,* and several PA structural genes, including *VviLAR1*, *VviLAR2,* and *VviANR* (Huang et al., 2014). Recently, we demonstrated that VviMYB86 enhances PA accumulation *via* the opposite regulations of the activities of PA and anthocyanin pathways (Cheng et al., 2021). bHLH transcription factors, also known as MYCs, are divided into 15 ∼ 25 subfamilies in plants (Li et al., 2006; Carretero-Paulet et al., 2010). This group of proteins contains a highly conserved domain of approximately 60 amino acids, forming the basic region and the helix–loop–helix (HLH) region (Li et al., 2006). The basic region contains an HER motif (His-Glu-Arg), which is mainly responsible for recognizing and binding the E-box (CANNTG) element or its variant G-box (CACGTG) in promoters of target genes (Heim et al., 2003; Hichri et al., 2010). The HLH region carries two amphipathic α-helices separated by a diverged loop and is responsible for the protein dimerization and the regulator complex formation (Carretero-Paulet et al., 2010). bHLH transcription factors regulate multiple biological processes in plants, including plant growth and development, signal transduction, stress response, and secondary metabolism (Carretero-Paulet et al., 2010; Pires and Dolan, 2010; Zhao et al., 2019a; Xu et al., n.d.). A number of bHLH transcription factors have been found to regulate flavonoid biosynthesis in plants, such as *Freesia hybrida* (Li et al., 2016b), *Raphanus sativus* (Sun-Hyung et al., 2017), *Semen trigonellae* (Zhao et al., 2019a), and most of these bHLH transcription factors belong to the IIIf subgroup. In grapevine, at least 119 bHLH family proteins have been identified based on the genome sequence (Jaillon et al., 2007; Velasco et al., 2007), but only VviMYC1 and VviMYCA1 are shown to be associated with PA accumulation (Hichri et al., 2010; Matus et al., 2010). Until now, bHLH transcription factors that regulate *VviLARs* remain unknown, although E/G boxes are rich in *VviLAR1* promoter (Cheng et al., 2020, 2021).

Here, we investigated the role of a newly identified transcription factor VvibHLH93 in PA pathway through both *in vivo* and *in vitro* experiments. Evidence showed that VvibHLH93 negatively regulated PA biosynthesis in grapevine by mainly downregulating the expressions of the two *Vvi*LAR** genes. Our findings provide insights into the regulatory network of PA accumulation in relation to bHLH transcription factors.

## Materials and methods

### Plant materials and growth conditions

Grape materials of *Vitis. vinifera* L. cv. Cabernet Sauvignon were collected from the experimental vineyard at the Shangzhuang Experimental Station of China Agricultural University in Haidian district, Beijing (40°14′ N, 116°20′ E, altitude 49 m), during the 2019 growing season. Flowers, tendrils, stems, young and mature leaves, and berries of Cabernet Sauvignon at the indicated developmental stages were collected for gene expression analysis. Details on regional climate, grapevine cultivation, and sampling strategy were described in our previous publication (Cheng et al., 2021). The samples were snap-frozen in liquid nitrogen and stored at −80°C until required. Three biological replicates were taken for each sample. Cabernet Sauvignon berry skin-derived callus was cultured on solid B5 medium (3.21 g/L B5 basic medium, 30 g/L sucrose, 2.5 g/L acid-hydrolyzed casein, 0.2 mg/L kinetin (KT), 0.1 mg/L naphthalene acetic acid (NAA), 3.0 g/L plant gel; pH 5.9–6.0) and maintained in a growth chamber at 22°C in the dark. The subcultured period of grape callus was 25–30 days. Tobacco (*Nicotiana benthamiana*) used for dual-luciferase assay was grown in soil in the greenhouse under 16/8-h light/dark cycle at 23°C.

### Gene cloning and sequence analysis

Specific primers were designed based on the sequence data in the National Center for Biotechnology Information (NCBI) reference sequence database^1^ and grape genome database^2^ (Supplementary Table S1). The coding sequence (CDS) region of *VvibHLH93* was amplified from a normalized Cabernet Sauvignon complementary DNA (cDNA) library with specific primers by PCR (Mu et al., 2014) and was subcloned into pMD19-T vectors (TSINGKE, China) for sequencing validation.

The bHLH domain of VvibHLH93 was identified by the Inter-ProScan program.^3^ The theoretical isoelectronic point and molecular weight were calculated using ProtParam tool.^4^ The protein subcellular localization was predicted by using ProtComp.^5^ The amino acid sequences of VvibHLH93 and other bHLH homologs from different plant species retrieved by BLASTP program from NCBI were aligned using the DNAMAN6.0 software (Lynnon Corporation, United States). Phylogenetic analysis was conducted using Neighbor-Joining method of MEGA 7.0 software (Kumar et al., 2016).

### Subcellular localization and transactivation assay

To study the subcellular localization of VvibHLH93, the CDS of *VvibHLH93* without the stop codon was inserted into a pEZS-NL expression vector containing the green fluorescent protein (GFP) reporter gene. The recombinant plasmid pEZS-NL:VvibHLH93-GFP was transformed into *Allium cepa* epidermal cells using gene gun method described by Meng et al. (2020). The transactivation assay in yeast (*Saccharomyces cerevisiae*) was performed as previously reported with slight modifications (Wang et al., 2016). Briefly, the CDSs of *VvibHLH93*, *VviMYBPAR* (GenBank no. AB911341), *VviMYBC2-L1* (GenBank no. EU181425), were integrated into the pGBKT7 plasmid, respectively. Then, recombinant plasmids were, respectively, transformed into yeast host strain AH109, according to the manufacturer’s protocol (PT4087-1, Clontech, United States). Successful transformants were grown on corresponding SD medium (SD/Trp-, SD/Trp-/His-, SD/Trp-/His−/Ade-) for the transactivation detection. The primers used in this study were listed in Supplementary Table S1.

### RNA extraction, reverse transcription, and quantitative real-time PCR analysis

The procedure of RNA extraction, reverse transcription and quantitative real-time PCR (qRT-PCR) were described as our previously reported (Cheng et al., 2021). The setup of PCR reactions per gene comprised at least three biological replicates with three technical replicates each (at least nine values). *VviUbiquitin1* was used as the reference (Bogs et al., 2005). The data analysis followed the method described by Ruijter et al. (2009). The corresponding primers were listed in Supplementary Table S1.

### Yeast one-hybrid assay

Yeast one-hybrid (Y1H) assays were carried out with the Matchmaker Gold Yeast One-Hybrid System Kit (Clontech, United States) according to the manufacturer’s protocol (PT4087-1, Clontech, USA). Based on the distribution of predicted bHLH binding sites in *VviLAR1* promoter region, a short fragment of the *VviLAR1* promoter (753 bp to 794 bp upstream of ATG of *VviLAR1*) was subcloned into pAbAi vector to obtain pAbAi-P_VviLAR1_. The *VvibHLH93* CDS was inserted into pGADT7 vector to construct the prey-AD vector. Then, the linearized pBait-AbAi vector was transformed into Y1HGold. After determining the minimal inhibitory concentration of Aureobasidin A (AbA) for positive transformants, the prey-AD vector was transformed into the bait yeast strain. Successfully transformed yeast strains were grown on corresponding SD medium (SD/−Leu, SD/−Leu + AbA) for 3–5 days. The corresponding primers used were listed in Supplementary Table S1.

### Electrophoretic mobility shift assay

*VvibHLH93* CDS was subcloned into the pET-32a plasmid. The resulting construct was transformed into BL21 cells, and the subsequent expression and purification of the recombinant protein were carried out as previously described (Li et al., 2020). The 3′ end biotin hot probes and mutant probes were synthesized by Zoonbio Biotechnology, China. The cold probes were competitor probes without biotin label. The Electrophoretic mobility shift assays (EMSAs) were carried out with the LightShift^™^ Chemiluminescent EMSA Kit (Thermo Fisher Scientific, United States) according to the manufacturer’s protocol. The corresponding primers used were listed in Supplementary Table S1.

### Dual-luciferase assay in a tobacco transient expression system

*VvibHLH93* CDS was cloned into the pCAMBIA 1,301 vector to express the effector. The promoter fragments of *VviDFR*, *VviLAR1*, *VviLAR2*, *VviANS*, *VviANR*, and *VviUFGT* were sub-cloned into the pGreenII 0800-LUC vector as reporter, respectively. The corresponding primers used in this study were listed in Supplementary Table S1. Individual effector vector and recombinant reporter vector were transferred into *A. tumefaciens* EHA105, respectively. Each kind of reporter was mixed with the effector or empty pCAMBIA 1,301 vector (control) at a 1:1 (v:v) ratio, and then injected into tobacco leaves as described previously (Meng et al., 2020). Dual-luciferase assay was carried out by using Dual-Luciferase^®^ Reporter Assay System (Promega, United States). The firefly luciferase (LUC) and Renilla luciferase (REN) values were measured by a VICTOR^®^ Nivo^™^ Multimode Plate Reader (Promega, United States). The relative LUC activity was calculated as the ratio between LUC and REN activities. Six biological replicates were applied.

### *VvibHLH93* overexpression in grape callus

The full-length CDS of *VvibHLH93* was subcloned into the pCXSN vector, followed by the transformation of *A. tumefaciens* strain GV3101. The specific procedure of grape callus transformation was followed the methods previously described (Eck et al., 2006; Meng et al., 2020; Cheng et al., 2021). RNA was extracted from transgenic and wild-type (WT) callus and reverse-transcribed to cDNA as the template for gene expression analysis. Primers used in this section were listed in Supplementary Table S1.

### Determination of PA content

The visualization of PAs in grape callus was performed by *p*-dimethylaminocinnamaldehyde (DMACA) staining following the published methods (Li et al., 1996; Cheng et al., 2021). Three biological replicates were applied for each set of samples. The extraction and quantification of soluble and insoluble PAs were performed using the methods previously described (Yu et al., 2019). (+)-Catechin (Sigma, United States) and procyanidin B1 (Sigma, United States) were used as standards, respectively.

### Statistical analysis

Data in the bar plots were presented as the mean ± SD with at least three biologically independent replicates. Statistical analysis was conducted by two-tailed Student’s *t*-test using GraphPad Prism 8 (GraphPad Inc.).

## Results

### Identification of VvibHLH93

**Figure 2:**
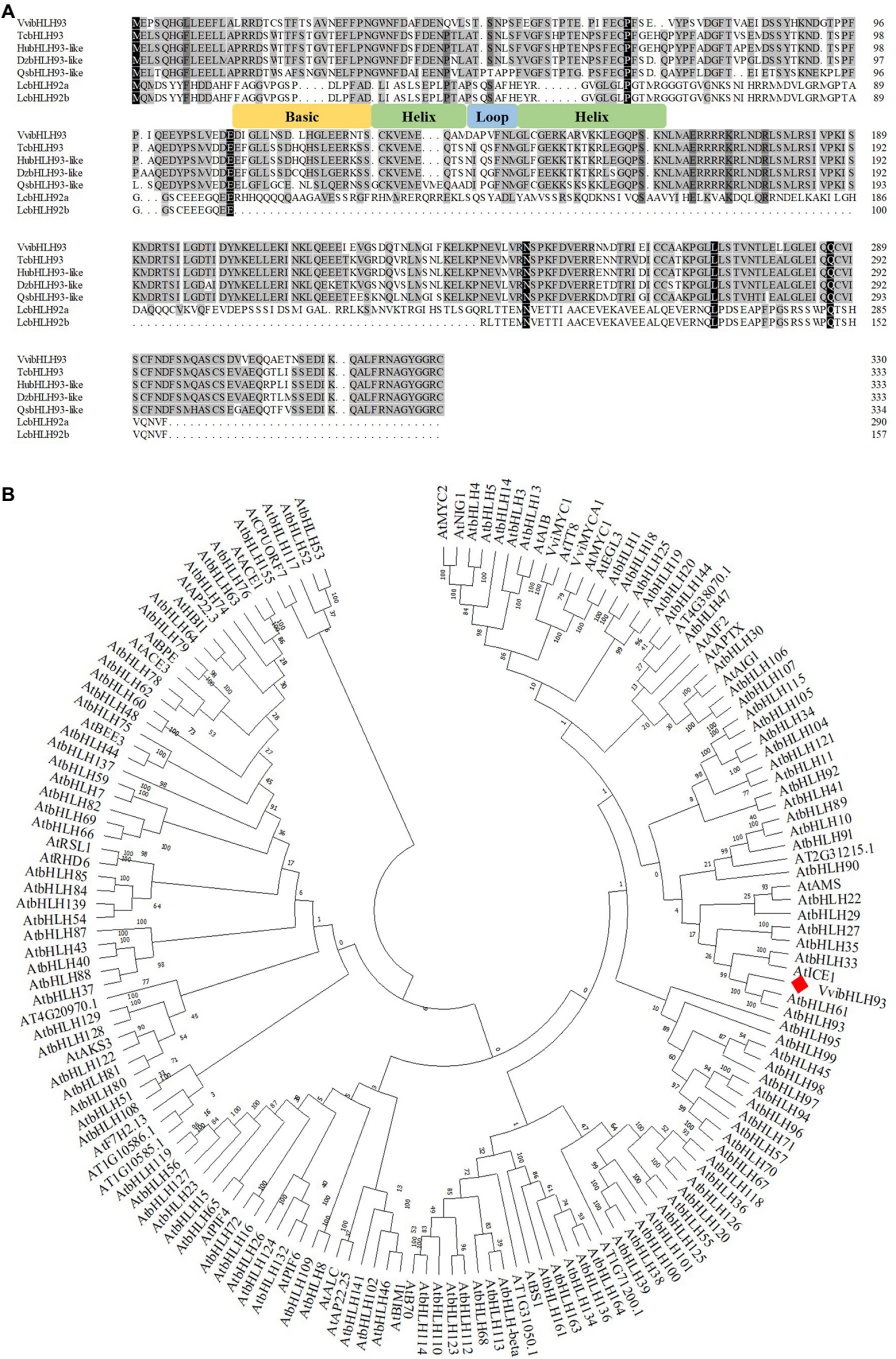
The analysis of VvibHLH93 amino acid sequence. **(A)** Alignment of the full-length amino acid sequences of VvibHLH93 and its homologs in other plants. GenBank accession numbers were as follows: VvibHLH93 (XP_002264407), TcbHLH93 (XP_007047694), HubHLH93-like (XP_021274415), DzbHLH93-like (XP_022740912), QsbHLH93-like (XP_023879689), LcbHLH92a (ATQ36622), LcbHLH92b (ATQ36623). Tc, *Theobroma cacao*; Hu, *Herrania umbratica*; Dz*, Durio zibethinus*; Qs, *Quercus suber*; Lc, *Leymus chinensis*; **(B)** Phylogenetic tree of bHLH proteins from *Vitis vinifera* and *Arabidopsis thaliana*. GenBank accession numbers were as follows: VviMYCA1 (NP_001267954) and VviMYC1 (NP_001268182). The protein sequences of all bHLH transcription factors from *A. Arabidopsis* were obtained from Plant Transcription Factor Database (http://planttfdb.gao-lab.org/family.php?sp=Ath&fam=bHLH). The analysis was performed with MEGA version 7 *via* the neighbor-joining method, with 1,000 bootstrap replicates.

Our previous studies show that the promoter of *VviLAR1* contains multiple E/G box elements, which are supposed to be the motifs for bHLH transcription factor recognition (Cheng et al., 2020, 2021). However, bHLH proteins that directly regulate *VviLAR1* remain unknown. By performing Y1H screening against the Cabernet Sauvignon berry cDNA library (Mu et al., 2014), we found the protein annotated as VvibHLH93 in grape genome database^6^ was a putative *VviLAR1* promoter binding protein. It was shown that the full-length *VvibHLH93* CDS amplified from Cabernet Sauvignon berry cDNA encoded 331 amino acids with a predicted molecular weight of 50.04 kDa and a calculated isoelectric point of 4.77. Analysis with Inter-ProScan program^7^ revealed that a conserved bHLH domain was located in the middle of VvibHLH93 sequence (Figure 2A). According to the phylogenetic tree constructed using bHLH protein sequences from *Arabidopsis thaliana* and grapevine (*Vitis vinifera*), VvibHLH93 was not in the same subclade with the known bHLH regulators of PA pathway (AtTT2, VviMYCA1, and VviMYC2), while it was highly in orthologous relationship to AtbHLH61 and AtbHLH93 of *A. thaliana* in the IIIB subgroup (Figure 2B).

### Subcellular localization and transcription activity of VvibHLH93

**Figure 3:**
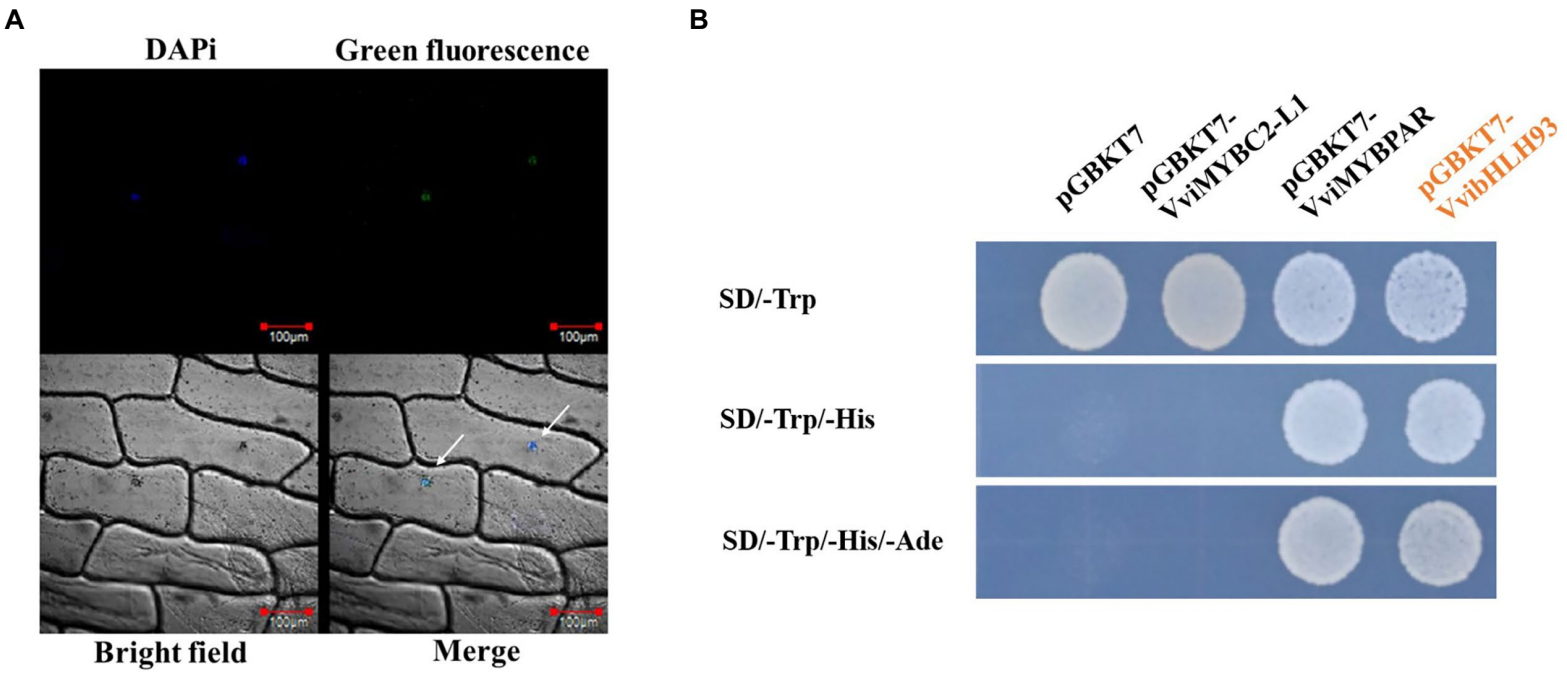
Subcellular localization and transcription activation of VvibHLH93. **(A)** Green fluorescence detection in onion epidermal cells transiently expressing a p35S:VvibHLH93-GFP construct. Bar: 100 μm. GFP, green fluorescence channel; Marker, Blue fluorescence channel, DIC: bright light channel; Merge, the GFP and DAPi overlap. Arrows indicate the sites of GFP and DAPi overlap. **(B)** Transcriptional activation by VvibHLH93 in yeast. The pGBKT7-MYBPAR plasmid was applied as a positive control. The pGBKT7-MYBC2-L1 and pGBKT7 plasmids were served as the negative controls. SD/−Trp: SD medium lacking tryptophan. SD/−Trp-His: SD medium lacking both tryptophan and histidine. SD/−Trp–His-Ade: SD medium lacking tryptophan, histidine, and adenine.

VvibHLH93 was predicted as a nuclear-localized protein using ProtComp algorithm.^8^ To examine the subcellular localization of VvibHLH93, the full-length CDS of *VvibHLH93* without the stop codon was fused with *GFP* and expressed in onion (*Allium cepa* L.) epidermal cells under the control of the *35S* promoter. Confocal microscopy analysis showed that the green fluorescence of VvibHLH93:GFP fusion protein was merged with the cell nucleus stained by DAPi (Figure 3A), indicating that VvibHLH93 may function as a transcription factor. To test the transactivation activity, the full-length CDS of *VvibHLH93* was subcloned into pGBKT7 vector to express VvibHLH93 fused with the GAL4-DNA binding domain (BD) in the yeast system. In parallel, yeast cells containing pGBKT7-VviMYBPAR was used as the positive control and that transformed with pGBKT7-VviMYBC2-L1 or the empty vector were served as the two negative controls. Yeast cells expressing GAL4 BD-VvibHLH93 or GAL4 BD-VviMYBPAR were able to grow on SD-Trp-His-Ade medium, whereas the negative control cells transformed with the pGBKT7 empty vector or pGBKT7-VviMYBC2-L1 were only able to grow on SD-Trp medium (Figure 3B). This means that VvibHLH93 processed transactivation activity.

### VvibHLH93 bound the E/G-box elements in the *VviLAR1* promoter

**Figure 4:**
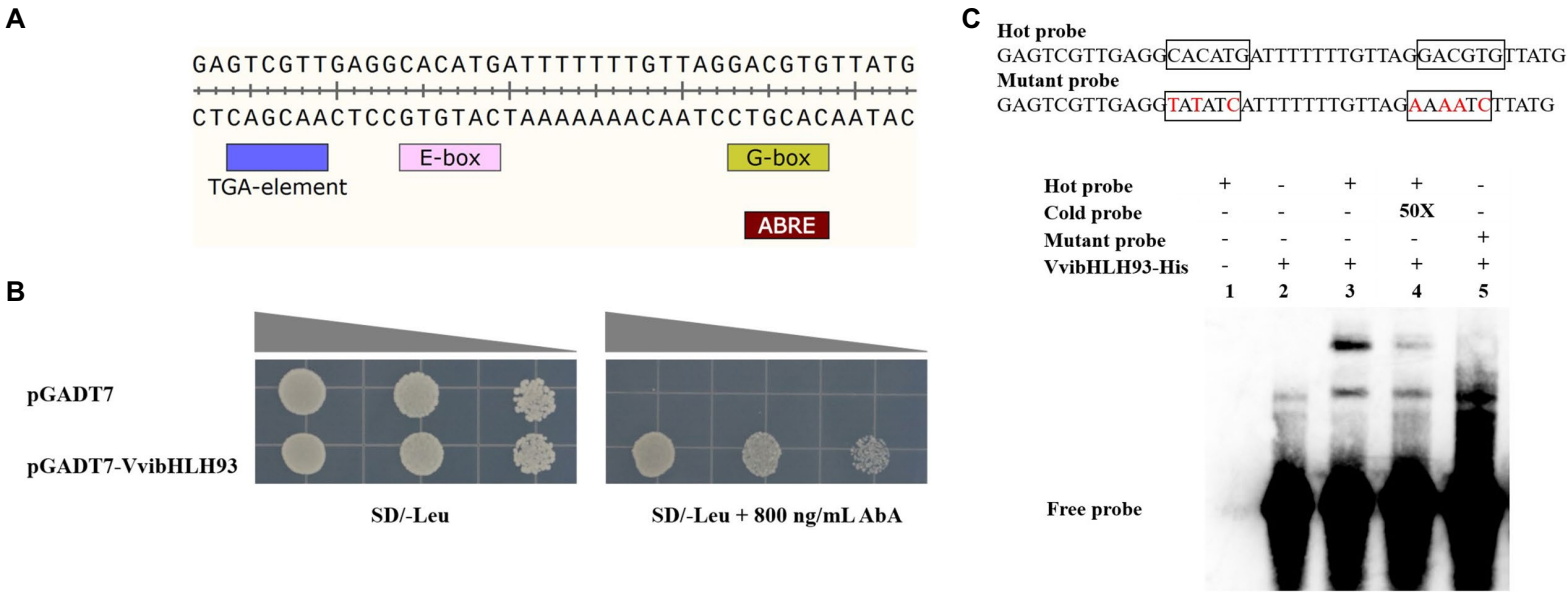
VvibHLH93 binds the promoter of *VviLAR1*. **(A)** The *cis*-element analysis in the sequence of partial *VviLAR1* promoter (the bait). TGA-element, MYC, G-box, and ABRE are predicted *cis*-acting elements and transcription factor binding sites on *VviLAR1* promoter. **(B)** The Y1H verification of the interaction between VvibHLH93 and *VviLAR1* promoter. From left to right, the OD of the bacterial solution are 0.05, 0.01, and 0.002. **(C)** EMSA assays of binding of VvibHLH93-His. The probe sequence marked by a black box is the proposed binding sites MYC and G-box on the *VviLAR1* promoter. The mutated bases are marked in red; The 3′ biotin-labeled fragments were the hot probes, and the unlabeled partial *VviLAR1* promoter fragments in 50-fold excess (50X) relative to the labeled native probes were the cold probes. “+” and “−” indicate the presence and absence of protein or probe, respectively.

In order to study the VvibHLH93 binding sites in the *VviLAR1* promoter, Y1H assay and EMSA were carried out. In Y1H assay, the pAbAi vector carrying two E/G-box (5′-CANNTG/CACGTG-3′) from the *VviLAR1* promoter (Figure 4A) and the pGADT7-VvibHLH93 recombinant vector served as the reporter and the effector, respectively. The Y1HGold yeast co-transformed with effector and reporter grew normally on the SD/−Leu medium supplied with 800 ng/ml AbA, whereas the yeast carrying the reporter and the pGADT7 empty vector could not (Figure 4B), indicating that VvibHLH93 binds the E/G-box-containing region in the *VviLAR1* promoter. The interaction of VvibHLH93 with the *VviLAR1* promoter fragment was further confirmed by EMSA using the biotinylated probe containing the corresponding tandem E/G-box motifs or its mutated form. A shifted DNA-protein complex band was clearly observed when the fusion protein of VvibHLH93 was incubated with probe (Figure 4C). In addition, the band signal was less abundant when 50-fold concentration of cold probe was added, and no shift was detected in the system only containing mutant probe and the recombinant VvibHLH93. This suggested that VvibHLH93 could directly bind the E/G-box site of the *VviLAR1* promoter.

### Expression patterns of *VvibHLH93* in grapevine

**Figure 5:**
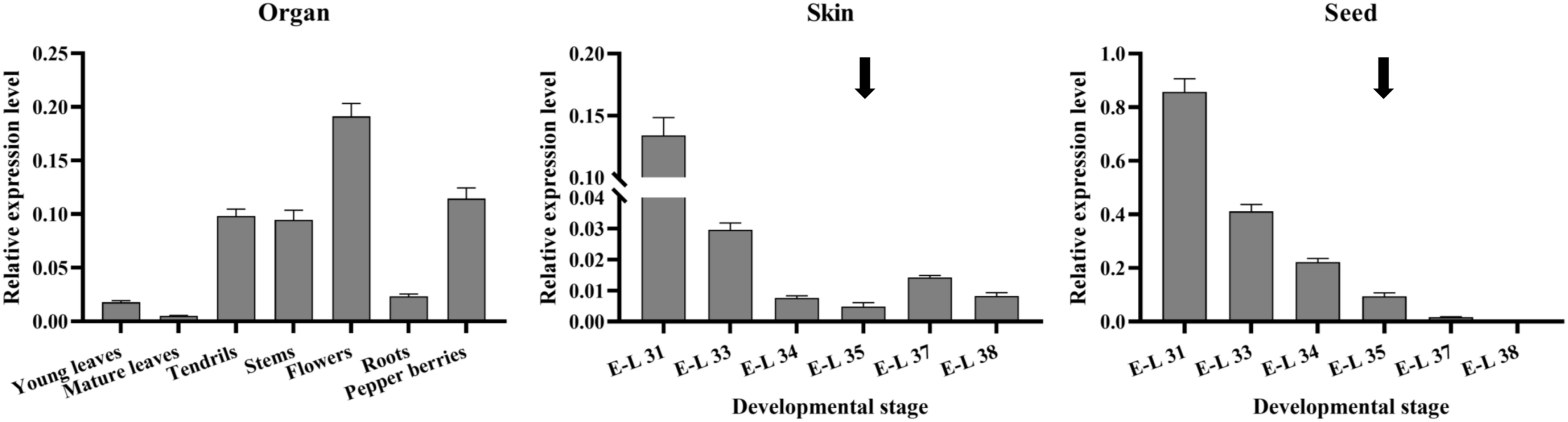
Expression profiles of *VvibHLH93* in different tissues at different developmental stages of grapes. *vvibHLH93* transcript level was determined by real-time quantitative PCR normalized relative to the expression of the expression of *VviUbiquitin1*. The data are shown as the mean ± SD (with *n* = 3 biologically independent replicates). The veraison period is indicated by the arrow. The left panel shows the expression profiles of *VvibHLH93* in different grapevine tissues. The middle panel shows the expression profiles of *VvibHLH93* at different developmental stages of grape skins. The right panel shows the expression profiles of *VvibHLH93* at different developmental stages of grape seeds.

qRT-PCR was performed to investigate the *VvibHLH93* expression patterns in different tissues of the grapevine (Figure 5). The results showed that *VvibHLH93* was mainly expressed in flowers, pepper berries (at the E-L 29 developmental stage), tendrils, and stems, while its transcript level was relatively low in young leaves, mature leaves, and roots. From the E-L 31 (pea-sized berry) onwards, skins and seeds were readily separated from grape berries. We further showed that the transcript level of *VvibHLH93* continuously decreased from E-L 31 to E-L 35 (veraison) in both berry skins and seeds. From E-L 35 to E-L 38 (harvest), *VvibHLH93* transcript level in seeds kept decreasing until not detectable, while that in berry skins increased slightly at E-L 37 and then dropped till the E-L 38. It is also noteworthy that *VvibHLH93* transcript level in seeds was ~5-fold higher than that in berry skins before E-L 37. Taken together, the expression of *VvibHLH93* is tissue-specific and berry development-dependent. During grape berry development, PAs biosynthesis starts from flowering and becomes inactive at version (Bogs et al., 2005). Considering this, the expression pattern of *VvibHLH93* is somehow linked with PA biosynthesis.

### The overexpression of *VvibHLH93* reduced PA content in grape callus

**Figure 6:**
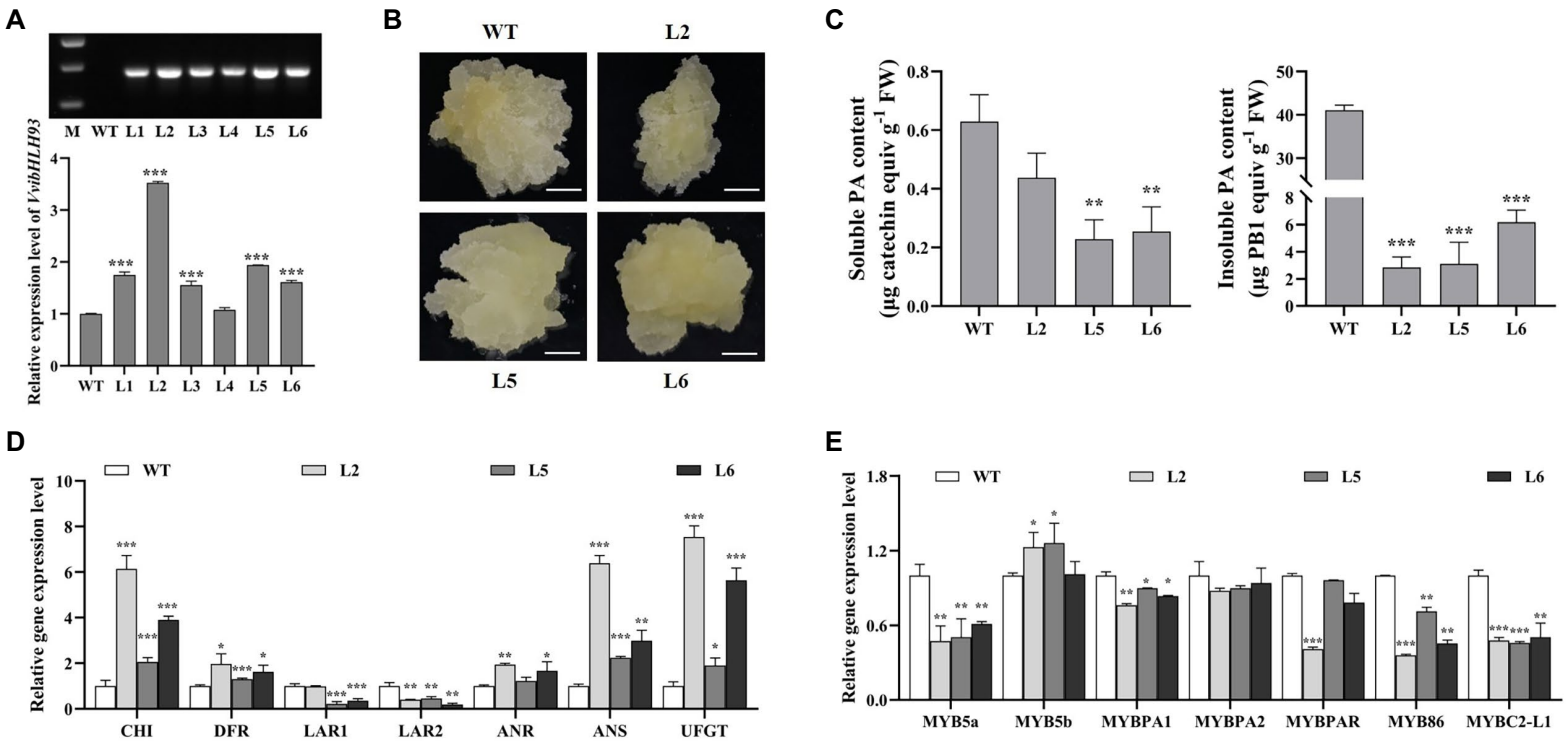
The overexpression of *VvibHLH93* repressed PA biosynthesis in grape callus. **(A)** Identification of transgenic callus. WT, wild-type grape callus; L1–L6, different transgenic lines. The upper panel shows RT-PCR for detection of hygromycin gene transcript in different grape calluses. M: D2000 marker. The lower panel shows the transcript level of *VvibHLH93* in WT callus and different transgenic lines (L1–L6). **(B)** Photos of grape callus cultured in their natural growth states. Scale bars indicate 1 cm. **(C)** The measurement of soluble and insoluble PAs in grape callus. FW, fresh weight. **(D)** Transcript levels of PA biosynthesis-related structural genes in grape callus. CHI, chalcone isomerase; FLS, flavonol synthase; DFR, dihydroflavonol-4-reductase; LAR, leucoanthocyanidin reductase; ANR, anthocyanidin reductase; ANS, anthocyanidin synthase; UFGT, UDP-glucose: flavonoid-3-*O*-glucosyltransferase. **(E)** Transcript levels of PA biosynthesis-related transcription factors in grape callus. For **(A,C–E)**, data are shown as the mean ± SD. (with *n* = 3 biologically independent replicates). Asterisks indicated significant differences relative to the control by two-tailed Student *t*-test, ^*^p < 0.05; ^**^p < 0.01; ^***^p < 0.001.

Previous studies showed that AtbHLH61 and AtbHLH93, the two homologs of VvibHLH93 (Figure 2B), are required for apical meristem function and flowering in *A. thaliana* (Sharma et al., 2016; Poirier et al., 2018). In contrast to grapevine, *A. thaliana* does not possess *LAR* (Lepiniec et al., 2006). This indicates that VvibHLH93 might possess the novel function in regulating PA pathway in grapevine besides the potentially similar functions as its homologs in *A. thaliana*. To further study the role of VvibHLH93 *in vivo*, the transgenic grape callus overexpressing *VvibHLH93* was generated. Three *VvibHLH93*-overexpressing transgenic grape callus lines (Line 2, Line 5, and Line 6) were selected for further analysis as the abundance of *VvibHLH93* transcript was increased by at least 1.5-fold compared with that of the WT (Figure 6A). The transgenic lines showed the white and loose morphological feature, which was similar with that of the WT callus (Figure 6B). Soluble PAs were isolated using 70% aqueous acetone from the transgenic and the WT callus and quantified using DMACA reaction. It was shown that all the transgenic lines produced slightly lower amounts of soluble PAs than the WT (Figure 6C). To measure insoluble PAs content in the callus, butanol/HCl analysis was further applied to the residues after 70% aqueous acetone extraction. Compared with the WT, overexpressing *VvibHLH93* resulted in at least 5-fold decrease of insoluble PAs (Figure 6C).

To elucidate the mechanism underlying the phenotypic changes observed in grape callus over-expressing *VvibHLH93*, the transcript levels of related genes in flavonoid pathway were further measured by qRT-PCR. As for PA structural genes, compared with the WT, the transcript levels of both *VviLAR1* and *VviLAR2* were largely decreased, while the expression of *VviANR* was slightly enhanced (Figure 6D). For other structural genes in the flavonoid pathway, transcript levels of *VviCHI*, *VviDFR*, *VviANS,* and *VviUFGT* in three transgenic lines were all higher than that in the WT callus (Figure 6D). Taken together, overexpressing *VvibHLH93* in the grape callus promoted the expression of most genes in the flavonoid pathway but inhibited *VviLARs* transcription. Although *VviUFGT* (the anthocyanin gene in competition with PA pathway) was upregulated by VvibHLH93, the transgenic callus did not turn red (Figure 6B), indicating the insufficient anthocyanin level for quantification analysis (Cheng et al., 2021). In addition, our previous work showed that VviLAR1 and VviLAR2 possess the same function in producing (+)-catechin PA starter unit, and a recent study suggested that silencing *VviLAR1* could sufficiently result in the lower concentration of PAs in the grape leaves and seeds (Yu et al., 2019; Robinson et al., 2021). This means that the reduced PA content in transgenic grape callus is mainly linked to the decreased transcript level of two *VviLAR*s rather than the product partitioning between PA and anthocyanin branches. Among the known regulators in the flavonoid pathway, *VviMYB5a*, *VviMYBPA1*, *VviMYB86,* and *VviMYBC2-L1* were significantly downregulated in all the transgenic lines, while the responses of *VviMYB5b* and *VviMYBPAR* to VvibHLH93 overexpression were shown to be dose-dependent (Figure 6E). The *VvibHLH93* transcript level was the highest in the Line 2, followed by that in the Line 5. Accordingly, *VviMYB5b* was induced in both the line 2 and the line 5, while *VviMYBPAR* was downregulated only in the line 2 (Figure 6E). These results further suggested that VvibHLH93 had a broad role for regulating flavonoid biosynthesis.

### Effect of VvibHLH93 on the promoter activities of the structural genes in PA and anthocyanin pathways

**Figure 7:**
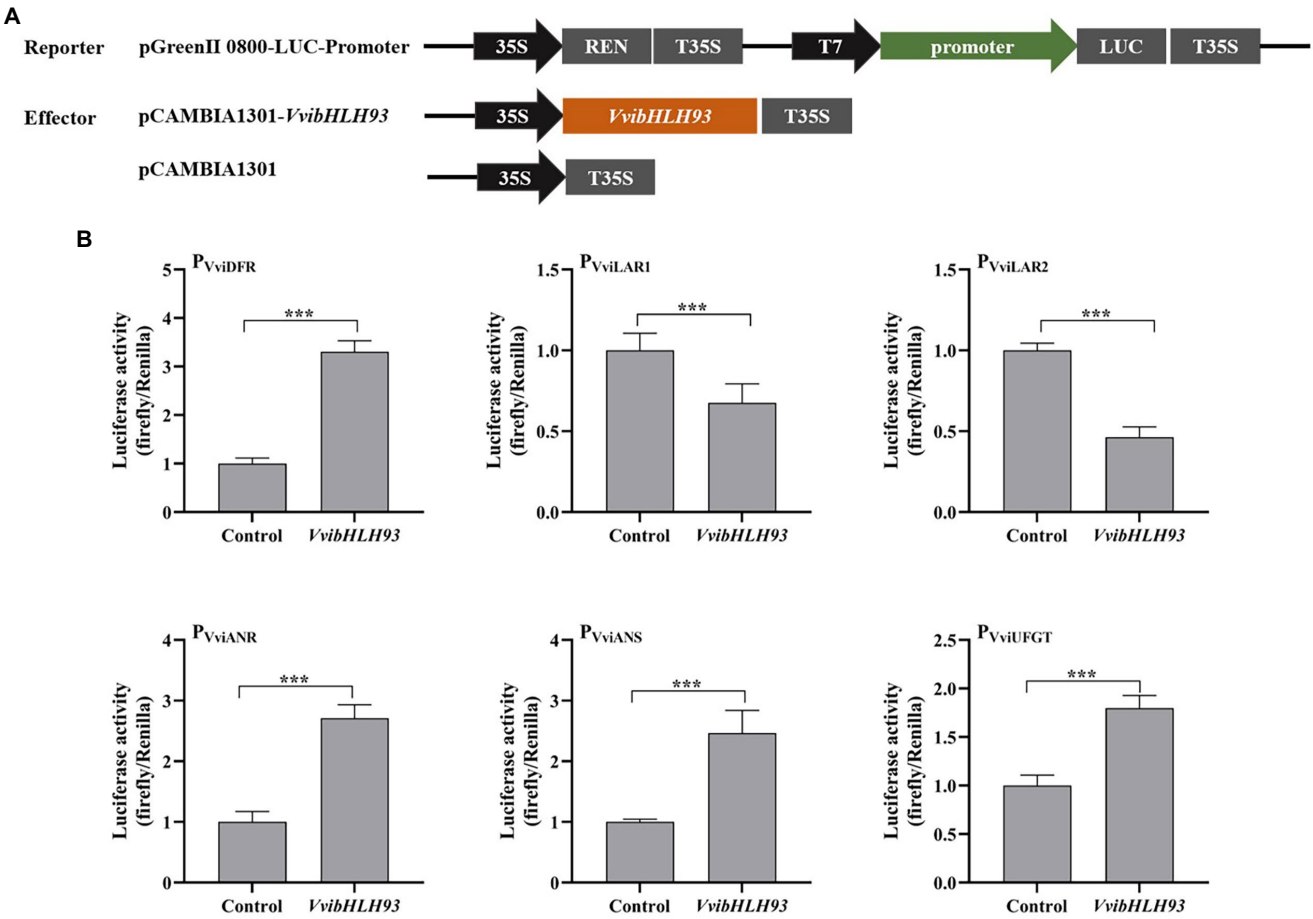
Effects of VvibHLH93 on the activities of the target promoters. **(A)** Schematic diagrams of vectors used for the dual luciferase assay; **(B)** Effects of VvibHLH93 on the activities of structural gene promoters of PA synthesis. Control indicated the activity of the promoter transfected with the empty vector (pCAMBIA1301). Each column represented means ± SD from four biological replicates. Asterisks indicated significant differences relative to the control by two-tailed Student’s *t*-test. ^***^p < 0.001.

To study whether VvibHLH93 directly regulated the expression of structural genes of grape PA and anthocyanin pathways, dual-luciferase assays were performed in *N. benthamiana* leaf transient expression system. Promoters of structural genes in PA biosynthetic and anthocyanin branches, including P_VviDFR_, P_VviLAR1_, P_VviLAR2_, P_VviANR_, P_VviANS_, and P_VviUFGT_ were cloned in our previous study (Cheng et al., 2021). The scheme for the construction of the reporter and effector vectors was demonstrated in Figure 7A. *VvibHLH93* and the corresponding promoters were co-transformed into *N. benthamiana* leaves followed by luciferase activity measurement (Figure 7A). The results revealed that *VvibHLH93* significantly repressed the activities of P_VviLAR1_ and P_VviLAR2_. In contrast, *VvibHLH93* enhanced the activities of P_VviDFR_, P_VviANR_, P_VviANS_, and P_VviUFGT_ (Figure 7B). These results were consistent with the data from the gene expression analysis in the transgenic grape callus (Figure 6D), further suggesting that VvibHLH93 suppressed *VviLAR*s transcription but promoted the expression of other structural genes involved in PA and anthocyanin synthesis, *via* direct affecting promoter activities of the structural genes along with regulating the transcript levels of flavonoid pathway transcription factors.

## Discussion

PAs derived from grape berries play crucial roles in the mouthfeel and color stabilization of red wines (Bogs et al., 2005). Here, we characterized the function of a new regulator VvibHLH93 in grape PA pathway. By using Y1H and EMSA approaches, we confirmed that VvibHLH93 could bind the tandem E/G boxes on the promoter of *VviLAR1.* Based on the putative motifs outside of bHLH conserved domains, the bHLH transcription factors of *A. thaliana* are divided into 25 subgroups (Heim et al., 2003; Carretero-Paulet et al., 2010). It is believed that bHLHs in IIIf-1 subgroup (such as AtTT8 from *A. thaliana* and PhAN1 from *Petunia hybrida*) regulate the general flavonoid pathway (Nesi et al., 2000; Spelt et al., 2000), while the IIIf-2 subgroup bHLH transcription factors (e.g., CpbHLH1 from *Chimonanthus praecox*, AtGL3 from *A. thaliana* and MdbHLH33 from *Malus* × *domestica*) only regulate the anthocyanin biosynthesis and do not affect PA biosynthetic route (Ramsay et al., 2003; Espley et al., 2007; Zhao et al., 2020). In the IVd subgroup, LcbHLH92 from *Leymus chinensis*, a homolog of *A. thaliana* bHLH92, is a negative regulator of the flavonoid biosynthesis. Overexpression of *LcbHLH92* in *A. thaliana* significantly inhibited the transcription of *DFR* and *ANS* in leaves and seeds, resulting in decreased anthocyanin and PA levels in both tissues (Zhao et al., 2019b). Phylogenetic analysis showed that VvibHLH93 was in close homolog relationship to *A. thaliana* AtbHLH61 and AtbHLH93, which belong to the IIIB subgroup (Poirier et al., 2018). The simultaneous knock-out of *AtbHLH61* and *AtbHLH93* results in the delay of flowering in *A. thaliana* (Poirier et al., 2018). In the present study, we showed that the contents of soluble and insoluble PAs were both decreased in *VvibHLH93*-overexpressing grape callus compared with that in the WT, revealing that IIIB subgroup bHLH proteins may possess an additional function in regulating PA biosynthesis.

Overexpressing *VvibHLH93* in grape callus could downregulate the PA negative regulator *VviMYBC2-L1* as well as a series of PA positive regulators, including *VviMYB5a*, *VviMYBPA1,* and *VviMYB86*. Given that VviMYBC2-L1 targets a series of positive regulators and structural genes in the PA pathway (Huang et al., 2014), it was reasonable to observe the increased transcript levels of *VviDFR*, *VviANS*, and *Vv/ANR* in the transgenic callus. We recently found that VviMYB86 down-regulates the expression of *VviANS* and *VviUFGT* and promotes that of *VviLAR1* and *VviLAR2* (Cheng et al., 2021), which could further explain the opposite responses of anthocyanin genes and *VviLAR*s to *VvibHLH93* overexpression in the grape callus. Additionally, *VviMYB5b* was shown to be a positive regulator of both PA and anthocyanin pathway (Deluc et al., 2008) but its transcript level was not increased in all of transgenic callus, suggesting that this transcription factor might not mainly involved in the upregulation of the structure genes in the current grape callus system. Consistent with the results from grape callus, the dual-luciferase assay suggested that VvibHLH93 also enhanced the promoter activities of the structural genes responsible for (−)-epicatechin-type PA and anthocyanin biosynthesis but weakened that of both *VviLAR1* and *VviLAR2* in (+)-catechin-type PA pathway, though VvibHLH93 only exhibited the transactivation activity in the yeast system. In *A. thaliana*, AtTT8 (a bHLH regulator) alone not only activates the promoters of several flavonoid structural genes but also recruits the negative regulator MYB2 to inhibit the promoter activities of these genes (Jun et al., 2015). Considering this, VvibHLH93 may regulate *VviLAR1* expression in a similar manner as AtTT8. We previously showed that an E-box is also existed in the *VviLAR2* promoter (Cheng et al., 2020) and therefore speculated that VvibHLH93 might also bind *VviLAR2* promoter. However, as the self-activation of *VviLAR2* promoter was still observed even though the AbA concentration was set as high as 1,000 ng/ml, the direct interaction between VvibHLH93 and *VviLAR2* promoter remained to be further verified by other approaches. For years, VviLARs functions are mainly characterized by biochemical approach *in vitro* and the ectopic expression in model plants (Bogs et al., 2005; Pfeiffer et al., 2006; Yu et al., 2019). Until recently, the extent that VviLAR1 contributes to PA accumulation in grapevine has been elucidated. Silencing *VviLAR1* in grapevine leads to a significant lower concentration of PAs in the leaves and seeds but not in berry skins, which is consistent with the spatiotemporal expression pattern of *VviLAR1* (Robinson et al., 2021). Thus, the decreased PA content in *VvibHLH93* overexpressing grape callus was more likely due to the reduced levels of the two LAR proteins, and the increased transcript levels *VviDFR*, *VviANR,* and *VviANS* were not able to rescue the decreased PA content *via* (−)-epicatechin-type PA biosynthesis in the transgenic grape callus.

In grape berry-related tissues, PA biosynthesis starts from the formation of the flower and is almost inactive in both grape berry skins and seeds at version (Bogs et al., 2005). It is worth noting that the expression pattern of *VvibHLH93* was positively correlated with the PA accumulation, although it was shown to be a negative regulator of PA biosynthesis. As the homologs of VvibHLH93 in *A. thaliana* regulate the flowering (Poirier et al., 2018), it was possible that VvibHLH93 also possesses the function in plant development based on the gene expression pattern. Deluc et al. showed that overexpressing *VviMYB5a* in tobacco caused a delay in anther dehiscence (Deluc et al., 2006) and we found that VvibHLH93 largely downregulated *VviMYB5a in vivo*. This suggests that VvibHLH93 may also participate in the maintenance of reproductive process possibly by preventing the over-accumulation of PAs and other relevant polyphenols. Taken together, our findings enrich the regulatory mechanism of the PA biosynthesis in grapes, and provide new insights into the role of IIIB subgroup bHLH proteins.

## Data availability statement

The datasets presented in this study can be found in online repositories. The names of the repository/repositories and accession number(s) can be found in the article/Supplementary material.

## Author contributions

KY, CD, and JW conceived and guided the experiments. JC and YS performed the experiment. JC analyzed the data and wrote the original draft. KY and CD edited the final manuscript. All authors contributed to the article and approved the submitted version.

## Funding

This research was funded by the National Natural Science Foundation of China (grant U20A2042) and Natural Science Foundation of the Jiangsu Higher Education Institutions of China (22KJB210012).

## Conflict of interest

The authors declare that the research was conducted in the absence of any commercial or financial relationships that could be construed as a potential conflict of interest.

## Publisher’s note

All claims expressed in this article are solely those of the authors and do not necessarily represent those of their affiliated organizations, or those of the publisher, the editors and the reviewers. Any product that may be evaluated in this article, or claim that may be made by its manufacturer, is not guaranteed or endorsed by the publisher.

## Footnotes

^1^ https://www.ncbi.nlm.nih.gov/refseq/

^2^ http://genomes.cribi.unipd.it/grape/

^3^ http://www.ebi.ac.uk/Tools/pfa/iprscan/

^4^ http://web.expasy.org/protparam/

^5^ http://linux1.softberry.com/

^6^ http://genomes.cribi.unipd.it/grape/

^7^ http://www.ebi.ac.uk/Tools/pfa/iprscan/

^8^ http://linux1.softberry.com/

## Abbreviations

ANR: Anthocyanidin reductase
ANS: Anthocyanidin synthetase
BD: Binding domain
cDNA: Complementary DNA
CDS: Coding sequence
DFR: Dihydroflavonol 4-reductase
DMACA: *p*-Dimethylaminocinnamaldehyde
EMSA: Electrophoretic mobility shift assay
LAR: Leucoanthocyanidin reductase
NCBI: National Center for Biotechnology Information
PAs: Proanthocyanidins
P_VviANR_: *VviANR* promoter
P_VviANS_: *VviANS* promoter
P_VviDFR_: VviDFR promoter
P_VviLAR1_: VviLAR1 promoter
P_VviLAR2_: *VviLAR2* promoter
P_VviUFGT_: *VviUFGT* promoter
qRT-PCR: Quantitative real-time PCR
UFGT: UDP-glucose: flavonoid-3-*O*-glucosyltransferase
WT: Wild type
Y1H: Yeast one-hybrid
